# Age Differences in the Variability and Distribution of Sleep Spindle and Rapid Eye Movement Densities

**DOI:** 10.1371/journal.pone.0091047

**Published:** 2014-03-05

**Authors:** Kevin R. Peters, Laura B. Ray, Stuart Fogel, Valerie Smith, Carlyle T. Smith

**Affiliations:** 1 Department of Psychology, Trent University, Peterborough, Ontario, Canada; 2 Brain & Mind Institute, Western University, London, Ontario, Canada; 3 Department of Psychology, Western University, London, Ontario, Canada; University of Pennsylvania, United States of America

## Abstract

The present study had two main objectives. The first objective was to compare the sleep architecture of young and older adults, with an emphasis on sleep spindle density and REM density. The second objective was to examine two aspects of age differences that have not been considered in previous studies: age differences in the variability of sleep measures as well as the magnitude of age differences in phasic events across the distribution of values (i.e., at each decile rather than a single measure of location such as the mean or median. A total of 24 young (mean age = 20.75±1.78 years) and 24 older (mean age = 71.17±6.15 years) adults underwent in-home polysomnography. Whole-night spindle density was significantly higher in young adults than older adults. The two age groups did not differ significantly in whole-night REM density, although significant increases in REM density across the night were observed in both age groups. These results suggest that spindle density is more affected by age than REM density. Although age differences were observed in the degree of absolute variability (older adults had significantly larger variances than young adults for sleep efficiency and time spent awake after sleep onset), a similar pattern was also observed within the two age groups: the four sleep measures with the lowest degrees of relative variability were the same and included time spent in REM and Stage 2 sleep, total sleep time, and sleep efficiency. The distributional analysis of age differences in sleep spindle density revealed that the largest age differences were initially observed in the middle of the distributions, but as the night progressed, they were seen at the upper end of the distributions. The results reported here have potential implications for the causes and functional implications of age-related changes in sleep architecture.

## Introduction

Age differences in sleep architecture are well documented [1]. At the macroarchitecture level, a large meta-analysis of young and older adults revealed that age was significantly and negatively correlated with total sleep time (TST), sleep efficiency (SE), the percentage of time spent in slow-wave-sleep (SWS), and rapid eye movement (REM) sleep, and REM latency [2]. On the other hand, age was significantly and positively correlated with sleep onset latency (SOL), the amount of wakefulness after sleep onset (WASO), and the percent of time spent in stages 1 and 2. Thus, as adults get older their sleep tends to become lighter and more fragmented.

Age differences in sleep microarchitecture have also been reported [3]. The goal of the present study was to investigate age differences in two particular aspects of sleep microarchitecture: Stage 2 sleep spindle density and REM density. Briefly, sleep spindles are discrete EEG waveforms in the 12 to 16 Hz frequency range that occur during NREM sleep, being particularly prominent during Stage 2 sleep. REMs are conjugal eye movements that occur during REM sleep. Because the number of sleep spindles and REMs is dependent upon the time that individuals spend in these sleep stages, researchers often compute the density of these phasic events as the number of events per minute of a particular sleep stage.

Previous studies have found that the number or density of sleep spindles is reduced in older adults compared to younger adults [4]–[9], and the magnitude of this decrease seems to be most apparent at anterior regions [10]. Age-related decreases in spindle density have been reported for both slow (11–13 Hz) and fast (13–15 Hz) spindles [8]. Sleep spindle density increases across the night in young adults but not older adults [6], [9], [10]. Thus, although older adults tend to have more Stage 2 sleep than younger adults, the density of sleep spindles in this stage is markedly reduced in older adults, and this difference is seen in both whole night analyses as well as analyses that look at the density of sleep spindles across the night.

It has been speculated that age differences in spindle density may be due to decreases in thalamo-cortical and intra-cortical integrity [3], [6], [7], or to decreased levels of melatonin [11], [12]. There are two main hypotheses regarding the functional implications of age-related spindle changes. First, due to their possible role in promoting and/or maintaining sleep [13]–[15], the observed age differences in spindle density may explain the difficulties that many older adults have maintaining efficient and consolidated periods of sleep, particularly towards the end of the night [4], [6], [7]. Thus, reduced spindles may contribute to poorer sleep maintenance in older adults. It has also been suggested that age differences in spindle density are related to age differences in intellectual or cognitive functioning [16], [17]. The support for each of these functional hypotheses to date, however, has been limited.

In contrast to the consistent pattern of age-related reductions in spindle density, the picture for REM sleep and REM density is less clear, and requires further investigation. Several investigators have reported no differences in REM activity or density in older adults compared to young adults [5], [18], [19]. In contrast, significant reductions in REM activity or density in older adults has been reported in at least two studies [20], [21].

In terms of time-of-night effects, one study has reported significant increases in REM density across the night in young but not older adults [19]. Other studies have reported that most of the change across the night for REM parameters is limited to the first one or two REM periods. Feinberg and colleagues [5] observed that REM activity increases significantly from the first to the second REM period in both young and older adults, but there were no further significant changes from that point onwards. Darchia et al. [22] reported a significant age by cycle interaction for REM density that was driven primarily by a pattern of reduced REM density in the first REM period followed by elevated, but quite stable, REM densities afterwards in young participants; this pattern was not observed in the older participants. Thus, REM density seems to increase across the night in young adults, with the majority of this increase occurring early in the night.

In studies reporting age differences in REM density, it has been speculated that these differences are due to degenerative changes, such as the aging of brain areas involved in eye movement control or to the reduction of acetylcholine, a neurotransmitter involved in both REM sleep and cognition [20]. This link between REM density, aging, and cognition has received some limited support [5], [23], [24].

In our review of the literature on age differences in sleep spindle density and REM density, we have made two interesting observations. First, surprisingly, few studies have investigated age differences in sleep spindle and REM density in the same study. Accordingly, it is difficult to interpret age differences in sleep spindle and REM density when each phasic event is being assessed in separate studies, and thus in potentially separate samples of participants. These studies do not allow us to address the question of whether age differences in these two types of phasic events are related or whether they are independent (e.g., do older adults with reduced spindle densities also have reduced REM densities?). The answer to this question will help shed light on the causes and functional implications of age differences in these two types of phasic events. Thus, we sought to examine these two types of phasic events in the same participants to address this important question.

We have found only one study that has examined the density of both spindles and REMs. Feinberg [5] reported age differences in spindle density but not REM density. The present study differs from that of Feinberg in several important ways. Regarding spindles, Feinberg counted spindles from only 50 epochs of SWS across the night. In contrast, we counted all spindles from Stage 2 across the night. For REM density, Feinberg counted the number of epochs during REM sleep that contained one or more eye movements, whereas we counted the number of actual eye movements during REM sleep. Thus, we feel that our approach provides more detailed indices of spindle and REM density.

The second observation that became apparent to us with respect to the literature on age differences in sleep microarchitecture is that virtually all studies have focused on only one aspect of age differences: the mean age differences in these phasic events. Although age differences in the average levels of sleep spindles and REMs are important to document, we also felt that it would be useful to examine age differences in the variability of these phasic events. Age-related increases in variability have been documented for a number of physiological and cognitive variables [25], including phasic events during sleep [3]; however, there has been no real attempt to systematically examine such age differences for sleep architecture. How variable are sleep spindle and REM densities compared to other aspects of sleep architecture, and are these patterns comparable across the two age groups? In this study, we have examined inter-individual variability (i.e., differences between individuals) in two ways. First, we examined age differences in absolute variability (i.e., is the dispersion of data for a given measure more variable in the young group than in the older group)? Second, we examined the degree of variability of each sleep measure relative to other sleep measures separately in the young and older groups (e.g., how variable is spindle density compared to other measures of sleep architecture such as REM density, TST, SWS, etc.)?

In addition, age differences in the mean level of a particular phasic event do not necessarily imply that the same magnitude of effect is present across the entire distribution of values [26], [27]. In other words, is the magnitude of difference between the two means the same for individuals with lower levels and higher levels of these phasic events? Addressing these questions about variability and the magnitude of age differences across the entire distribution of values will help shed light on the causes and functional implications of age differences on sleep microarchitecture. The answers to these questions might also help to explain why some adults are able to age more gracefully than others in terms of their sleep architecture and cognitive functioning.

In summary, the present study sought to address three main gaps in the literature. First, we examined sleep spindle and REM density data from the same participants. Second, we systematically examined age differences in the variability of sleep spindle and REM density values in comparison to other aspects of sleep architecture. Third, we have extended upon previous studies that have reported on age differences in the mean level of sleep spindle and REM density values by examining age differences throughout the entire distribution of values (e.g., are age differences present at the low, middle, and high ends of the distributions?).

We predicted that whole night Stage 2 spindle density would be greater in young adults than in older adults. We also predicted that Stage 2 spindle density would show a significant increase across the night in young but not older adults. The literature on age differences in whole night REM density is less clear so we did not make any specific predictions about this phasic event. Regarding time of night effects for REM density, however, the literature suggests that REM density increases in young adults and most of this increase is limited to the early part of the night. Time of night effects for REM density in older adults is less clear. We therefore predicted that REM density would increase across the night in young adults but not older adults. Given the novel and exploratory nature of investigating age-related differences in the variability of sleep parameters and the magnitude of effect across the entire distribution of sleep spindle and REM densities we did not make any specific predictions beyond the general expectation that patterns of variability in sleep spindles and REMs across the night might differ in young and older subjects.

## Materials and Methods

### Participants

A total of 24 healthy young adults (12 female; mean age = 20.75±1.78 years, range: 17 to 24) and 24 healthy older adults (12 female; mean age = 71.17±6.15 years, range: 60 to 85) participated in this study. The young adults were all students and received course credit for participating in the study. The older adults were recruited from the surrounding local community. All potential participants reported themselves to be in good health, were non-smokers, avoided excessive alcohol and caffeine consumption, and had no history of depression or chronic pain. Participants completed an interview/questionnaire that comprised questions about sleep patterns/quality to ensure that they kept a regular sleep-wake schedule, did not experience difficulty falling asleep or staying asleep, did not experience excessive daytime sleepiness, or have difficulty breathing while asleep. We also excluded individuals with medical conditions, or those taking medications, that might interfere with sleep architecture or cognitive functioning. Symptoms of depression and signs of sleep disorders were screened using the Beck Depression Inventory [28] and the Sleep Disorders Questionnaire [29], respectively. We excluded participants who scored above 13 on the Beck Depression Inventory [30]. In the Statistical Analysis section below we describe our rationale for reporting the median (Mdn) as a descriptive measure of location and the median absolute deviation from the median (MAD) as a descriptive measure of scale. We also describe the Brunner-Munzel rank test for group differences and our rationale for using this test. The median score on the Beck Depression Inventory for the young group was 3.50 (MAD = 2.97; range: 0 to 13); in the older group it was 2.50 (MAD = 2.22; range: 0 to 12). This difference was not statistically significant, *p* = .246. Thus, none of the participants scored above the cut-off of 13 on the Beck Depression Inventory, and the two age groups did not differ significantly on this scale.

We used published cut-off scores for the Sleep Apnea (SA), Narcolepsy (NAR), Psychiatric Sleep Disorder (PSYC), and Periodic Limb Movement (PLM) subscales of the Sleep Disorders Questionnaire (SDQ) [29]. Cut-off scores for these subscales are provided separately for males and females. Table 1 contains these cut-off scores as well as the descriptive statistics and group differences on the SDQ subscales. As can be seen, none of the participants scored above the published cut-off scores for possible sleep disturbances, which increases our confidence that our participants did not have any of these sleep disorders. The two age groups did differ significantly, however, on several of the subscales. In both the female and male samples, older adults scored higher on the SA and PLM subscales than young adults. In addition, young males scored higher on the PSYC subscale than older males.

**Table 1:** Descriptive statistics and group differences on the Sleep Disorders Questionnaire.

		Young Group			Older Group			Test Statistic
Subscale	Cut-off Score	*Mdn (MAD)*	Range	*M_Rank_*	*Mdn (MAD)*	Range	*M_Rank_*	*W*
*Females*								
SA	32	14.50 (2.97)	6–23	8.21	22.00 (2.97)	16–27	16.79	4.65***
NAR	31	20.50 (3.71)	11–27	14.96	17.00 (4.45)	10–25	10.04	−1.82
PSYC	21	15.00 (5.93)	8–20	13.00	13.00 (2.97)	11–18	12.00	−0.32
PLM	21	12.50 (2.22)	10–21	9.46	16.00 (4.45)	11–20	15.54	2.40*
*Males*								
SA	36	19.00 (7.41)	12–27	7.90	27.00 (6.67)	20–33	15.75	4.13***
NAR	30	17.00 (2.97)	10–21	11.59	17.50 (3.71)	12–27	12.38	0.27
PSYC	19	16.00 (2.97)	12–19	15.68	13.00 (1.48)	9–17	8.63	−3.33**
PLM	21	11.00 (1.48)	9–20	8.09	16.00 (5.19)	11–21	15.58	3.55**

Notes: SA, sleep apnea; NAR, narcolepsy; PSYC, psychiatric sleep disorder; PLM, periodic limb movement; *Mdn*, median; *MAD*, median absolute deviation from the median; *M_Rank_*, mean of ranks; *W*, test statistic for the Brunner-Munzel rank test. Cut-off scores are from Douglass et al. (1994) [29].

*p<.05.

**p<.01.

***p<.001.

In addition, we performed a limited overnight screening for possible sleep disorders on the first night of recording. We examined EEG arousals in all participants to help identify significant periodic leg movements, respiratory events, or any other unusual events. Most of the older adults also underwent finger oximetry monitoring during the first night to help the scorer distinguish between signs of possible sleep disturbances as well as identify the severity of the respiratory events. All scoring was based on established American Academy of Sleep Medicine rules and guidelines for identifying and diagnosing sleep disorders [31], [32].

### Ethics Statement

All participants provided written informed consent and were financially compensated for their participation. The present study involved pooling data from several of our previous studies, each of which were approved by the Trent University Research Ethics Board.

### Polysomnographic Recordings

Suzanne (Tyco Healthcare Group LP, Mansfield, MA, USA) portable polysomnographic systems were used to perform in-home sleep recordings. Physiological data were recorded at a sampling rate of 120 Hz. EEG, EOG, and EMG recordings were taken using gold-plated electrodes. The EEG (C3 and C4) and EOG (left and right outer canthus of the eye) channels were recorded and referenced to the contralateral mastoid bones (A1 and A2). The EMG (submental chin muscles) channel was recorded as a bipolar derivation. Low- and high-pass software filters for the EEG and EOG were set at 0.03 Hz and 30 Hz, respectively. The low-pass software filter for the EMG channel was set at 10 Hz with no high-pass.

Sleep stages were scored in accordance to standard criteria [33] using Sandman software (Melville Diagnostics, Ottawa, ON, Canada). The following sleep macroarchitecture variables were computed: total sleep time (TST) in minutes, wakefulness after sleep onset (WASO) in minutes, sleep efficiency, the percent of TST spent in Stage 1, Stage 2, slow-wave-sleep (SWS; Stages 3 and 4 combined), and REM sleep. Due to the difficulties in precisely recording a ‘lights out’ time inherent with home recordings we do not report a sleep onset latency variable, nor do we report a total recording time variable.

An expert scorer visually identified both sleep spindles and REMs. Sleep spindles were counted from the C3 channel during epochs of Stage 2 sleep. C4 was used on occasion when the signal from the C3 channel was of poor quality. Spindles were in the 12 to 16 Hz frequency range, at least 0.5 sec in duration, and they had to resemble the typical fusiform spindle morphology. A duplicate EEG channel filtered for frequencies between 12 and 16 Hz was used to aid in spindle identification. This is particularly important to help identify spindles that overlap with slow events such as k-complexes or delta waves. Stage 2 spindle density was calculated by dividing the total number of sleep spindles during Stage 2 by the total number of minutes of Stage 2 sleep. REMs were counted from the EOG channels during epochs of REM sleep. Using the right or left EOG recording, eye movements had to be conjugal and exceed 15 µV in amplitude. Over the course of the studies included in the present data set, we have had two technicians scoring sleep architecture data. We assessed the inter-rater agreement between these two technicians on a random sample of 10 nights of data. The correlations between the two scorers on these sleep parameters were all above.93.

### Procedure

All interested participants were initially screened for eligibility (see details in Participants section above). In-home sleep recordings were performed for at least two consecutive nights. We used data obtained from the second night, after a night of acclimatization. On the evening of each night, a technician visited each participant’s home to prepare him or her for overnight polysomnography, and returned the following morning to pick up the recording unit and to download the recordings. Participants were asked to continue with their usual daily activities throughout the study.

### Statistical Analyses

All analyses were carried out using R open source statistical software [34], and data are available upon email request to the corresponding author. The assumptions of normality and homogeneity of variance were violated for a number of variables/group comparisons (detailed results for homogeneity of variance are reported in the Results section below). Accordingly, a robust nonparametric approach was adopted for all analyses [27], [35]. The median (Mdn) and the median absolute deviation from the median (MAD) were computed to provide descriptive information for the sleep architecture variables for each age group. To assess age differences in sleep architecture, the rank-based Brunner-Munzel method for independent groups [36] was used to compare the two age groups on total sleep time (TST) in minutes, sleep efficiency (SE), wakefulness after sleep onset (WASO), the percent of TST spent in Stage 1, Stage 2, Slow-Wave Sleep (SWS; Stages 3 and 4 combined), and REM sleep, spindle density, and REM density. The Brunner-Munzel test has been shown to be a more robust (i.e., better control over both type I and II errors) alternative to other nonparametric statistics such as the Wilcoxon-Mann-Whitney test [36].

The probability of superiority (PS) index was computed as an effect size measure for the age group comparisons [37], [38]. In this context, if one were to randomly select one young adult and one older adult from our samples, the PS is the probability that the young adult would have a higher score than the older adult. A PS value of 0.50 indicates no age difference. PS values between 0.50 and 1.0 indicate a greater probability of obtaining a higher score in favour of the young adult; PS values of 0.56, 0.64, and 0.71 correspond to the common guidelines proposed by Cohen for small, medium, and large effects, respectively [35]. PS values between 0 and 0.50 indicate a greater probability of obtaining a lower score in favour of the young adult; PS values of 0.44, 0.36, and 0.29 would correspond to small, medium, and large effect sizes respectively [39]. We also computed the 95% confidence interval for the PS: intervals that do not cross 0.50 are considered significant at the.05 alpha level. The Brunner-Munzel statistics and the PS effect size indices and confidence intervals were computed using the ‘WRS’ package for R [27] (http://dornsife.usc.edu/assets/sites/239/docs/Rallfun-v20).

In terms of variability, all of the analyses to be reported involve inter-individual variability (i.e., differences between individuals). In addition, we adopted two different approaches to examining variability. First, we adopted a much more conventional approach and examined age differences in absolute variability (i.e., is the dispersion of data for a given measure more variable in the young group than in the older group?). Age differences in absolute variability were assessed using the Bowne-Forsythe Levene type procedure [40], [41]. An ANOVA was performed on the residual scores for each participant (i.e., the absolute value obtained by subtracting each participant’s score from the median score for their group). P-values were computed in two ways: 1) the traditional approach of using the F-distribution, and 2) a bootstrapped version based on 2000 random samples (with replacement) [40], [41]. The process of bootstrapping has been described elsewhere [42], [43]. One of the main advantages of the bootstrap approach is that one does not rely on using theoretical distributions (i.e., normal or F-distribution), which may or may not be suitable, to derive confidence intervals or *p*-values. These statistics were computed using the ‘lawstats’ package for R (http://cran.r-project.org/web/packages/lawstat/index.html).

The second approach to examining inter-individual variability involved computing a coefficient of dispersion (CoD) to assess the degree of relative variability for each sleep variable within each age group. The CoD is simply the MAD divided by the Mdn, and is conceptually similar to the coefficient of variation (where the SD is divided by the mean). Given the non-normality of several of the variables, we felt that it would be inappropriate to use the more traditional coefficient of variation for this purpose. By taking into account the median, the CoD allows one to compare the degree of variability across variables that differ in their scales of measurement. In this way, we were able to compare the variability of a measure such as sleep spindle density in one age group to the variability of another measure such as total sleep time, both of which are measured on completely different scales of measurement. This analysis allowed us to determine if the degree of variability of sleep spindle and REM densities are in fact comparable to other aspects of sleep architecture, and whether these patterns are comparable across the two age groups. For example, it might be the case that the degree of variability of sleep spindles is much greater compared to other aspects of sleep architecture in the older age group but not in the young age group.

The night was divided into thirds to examine potential time-of-night effects. A rank-based split-plot analysis was carried out to assess 2(Age Group: Young, Older)×3(Time: First Third, Second Third, Last Third) models separately for spindle and REM density [44]. This method computes an ANOVA-Type Statistic (ATS) to assess the main effects of Age and Time, as well as their interaction. Analyses were carried out using the ‘nparLD package’ [44] for R (http://cran.r-project.org/web/packages/nparLD/index.html). PS effect size indices were also computed for appropriate two-way comparisons.

In order to compare the two groups across the entire distribution of values for sleep spindle and REM density, we followed the approach outlined by Wilcox [27]. First, we computed Harrell-Davis estimates, and associated standard errors, for each decile (0.10 through to 0.90) in each age group. Standard errors were computed using a bootstrap approach based on 200 random samples (with replacement). Difference scores for each decile were then computed and the 95% CI on these differences were estimated with a bootstrap approach based on 200 random samples (with replacement); 95% CIs not containing zero (i.e., no difference) were considered statistically significant. To provide some context, the group difference at the 0.50 decile is analogous to comparing the medians of the two groups.

To examine the association between sleep spindle and REM density, we computed Spearman rho correlation coefficients with bootstrapped 95% confidence intervals separately for young and older adults. Confidence intervals were generated based on 2000 random samples (with replacement) using the bias-corrected-and-accelerated method; 95% CIs that do not contain zero are considered statistically significant at the.05 alpha level. The bootstrap analysis was carried out using the ‘boot’ package for R (http://cran.r-project.org/web/packages/boot/index.html).

## Results

### Whole Night Sleep Architecture

Table 2 contains descriptive data, Brunner-Munzel test results, and effect size indices for the whole night sleep architecture data. The TST, SE, and SWS parameters were significantly greater in the young adults compared to the older adults, *p*<.001 for all. These effects are large, with PS values ranging from.79 to.89. Older adults spent significantly more time awake after sleep onset (WASO), in Stage 1 and Stage 2 sleep than young adults, *p*<.001 for all. Again, these effects were large, with PS values ranging from.19 to.08. The two age groups did not differ in the amount of time spent in REM sleep, *p*>.05, although the PS value of.62 indicates a medium-sized effect in favour of the young adults. As predicted, whole night sleep spindle density was significantly greater in young adults than in older adults, *p*<.001. The PS was.86, indicating a large effect. In contrast, REM density did not differ between the two age groups, *p*>.05. The PS value of.56 indicates a small effect.

**Table 2:** Age differences in whole night sleep architecture.

	Young Group		Older Group		Test Statistic	Effect Size	
	*Mdn (MAD)*	*M*_Rank_	*Mdn (MAD)*	*M*_Rank_	*W*	*PS*	95% CI
*Macroarchitecture*							
TST, min	486.50 (70.05)	31.56	397.75 (76.72)	17.44	4.49***	.79	.66 to .93
SE, %	96.25 (1.85)	33.88	84.15 (11.64)	15.13	8.31***	.89	.80 to .99
WASO, min	12.00 (7.78)	15.77	53.00 (53.37)	33.23	−6.91***	.14	.03 −.25
Stage 1, %	2.02 (1.47)	14.42	6.21 (2.23)	34.58	−9.96***	.08	.01 to .17
Stage 2, %	55.68 (8.40)	16.96	66.17 (7.22)	32.04	−5.00***	.19	.06 to .31
SWS, %	17.72 (8.90)	33.79	3.79 (5.10)	15.21	7.91***	.89	.79 to .99
REM, %	24.43 (5.73)	27.42	22.27 (4.88)	21.58	1.46	.62	.45 to .79
*Microarchitecture*							
Spindle Density	3.84 (1.43)	33.25	1.14 (0.83)	15.75	6.15***	.86	.74 to .99
REM Density	12.55 (2.82)	25.92	9.20 (4.00)	23.08	0.67	.56	.38 to .74

Notes: TST, total sleep time; SE, sleep efficiency; WASO, time awake after sleep onset; SWS, slow wave sleep; REM, rapid eye movement sleep; *Mdn*, median; *MAD*, median absolute difference from the median; *M*_Rank_, mean of ranks; *W*, test statistic for the Brunner-Munzel rank test; *PS*, probability of superiority (i.e., the probability of a young subject having a higher value than an older subject if one were to randomly select and compare one participant from each age group).

*p<.05.

**p<.01.

***p<.001.

Absolute variability was significantly greater in the older adults than in the young adults for SE, *F* = 11.78, *p* = .001 for both the traditional and bootstrap approaches, and WASO, *F* = 9.55, *p*<.003 for both approaches. The remaining group differences were not statistically significant, *p*>.16 for all.

**Figure 1:**
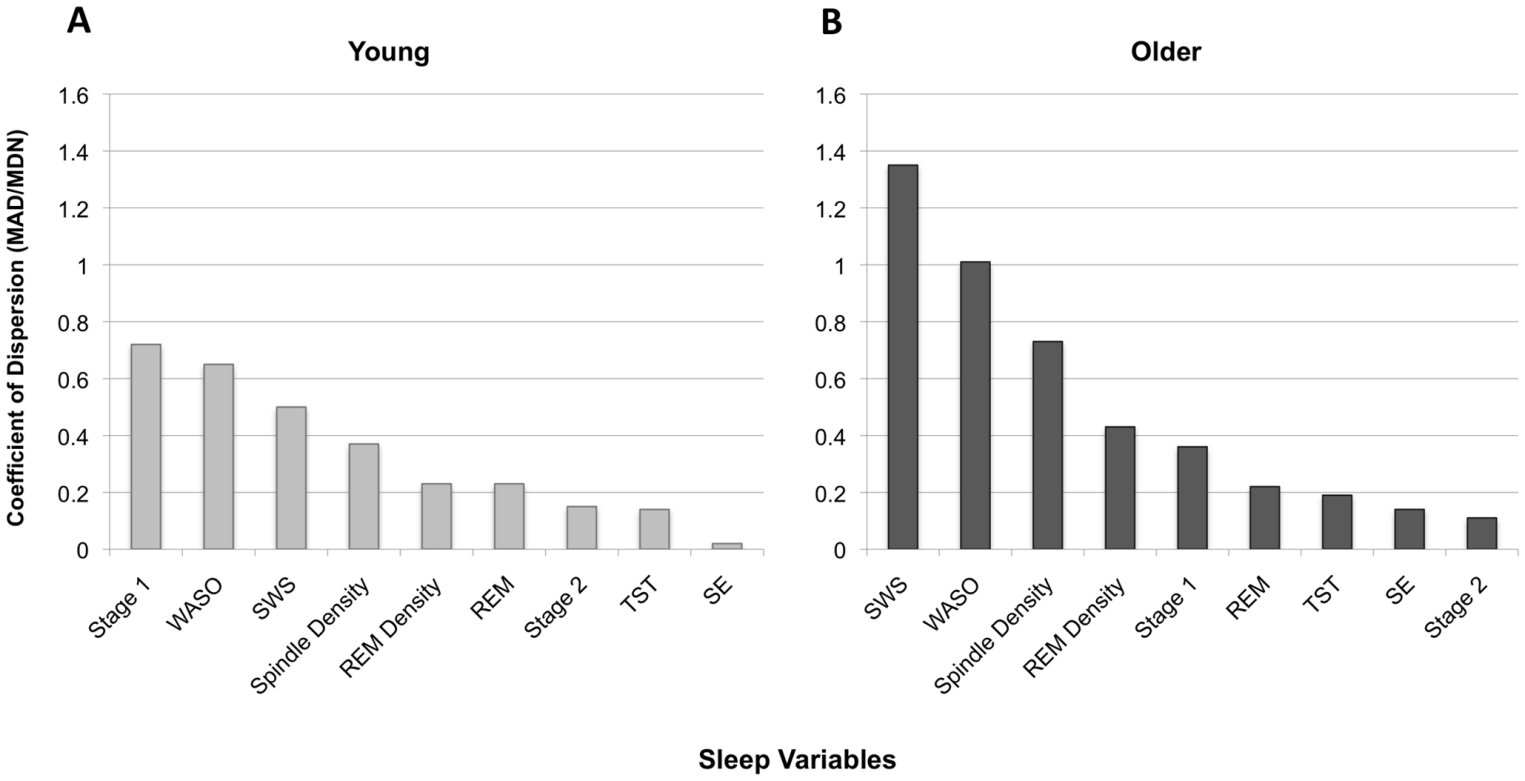
Relative variability of sleep measures. The Coefficients of Dispersion (CoD) are ordered from highest to lowest for Young adults (Panel A) and Older adults (Panel B). TST, total sleep time; SE, sleep efficiency; WASO, time awake after sleep onset; SWS, slow wave sleep; REM, rapid eye movement sleep; *MAD*, median absolute difference from the median; *Mdn*, median.

In terms of relative variability, the coefficients of dispersion (MAD/Mdn) are shown in Figure 1. To facilitate within-group comparisons, the coefficients of dispersion are ordered from largest to smallest for each age group. It is interesting to note the similarity in terms of overall pattern between the two age groups: In each group, the same four sleep variables (i.e., REM, Stage 2, TST, and SE) have the lowest CoD, and these all tend to be at or below the.20 level. Although the purpose of the relative variability analysis was not to compare the two age groups, it is interesting to note that the CoD for SE and WASO are considerably larger in the older group than in the young group, which is consistent with the results obtained from the Browne-Forsythe Levene type approach. There is some discrepancy, however, between the analyses of absolute and relative variability. Although the variances of the two age groups did not differ significantly with respect to SWS, sleep spindle density and REM density, the corresponding CoD values are considerably larger in the older group than in the young group.

### Time of Night Analysis

**Figure 2:**
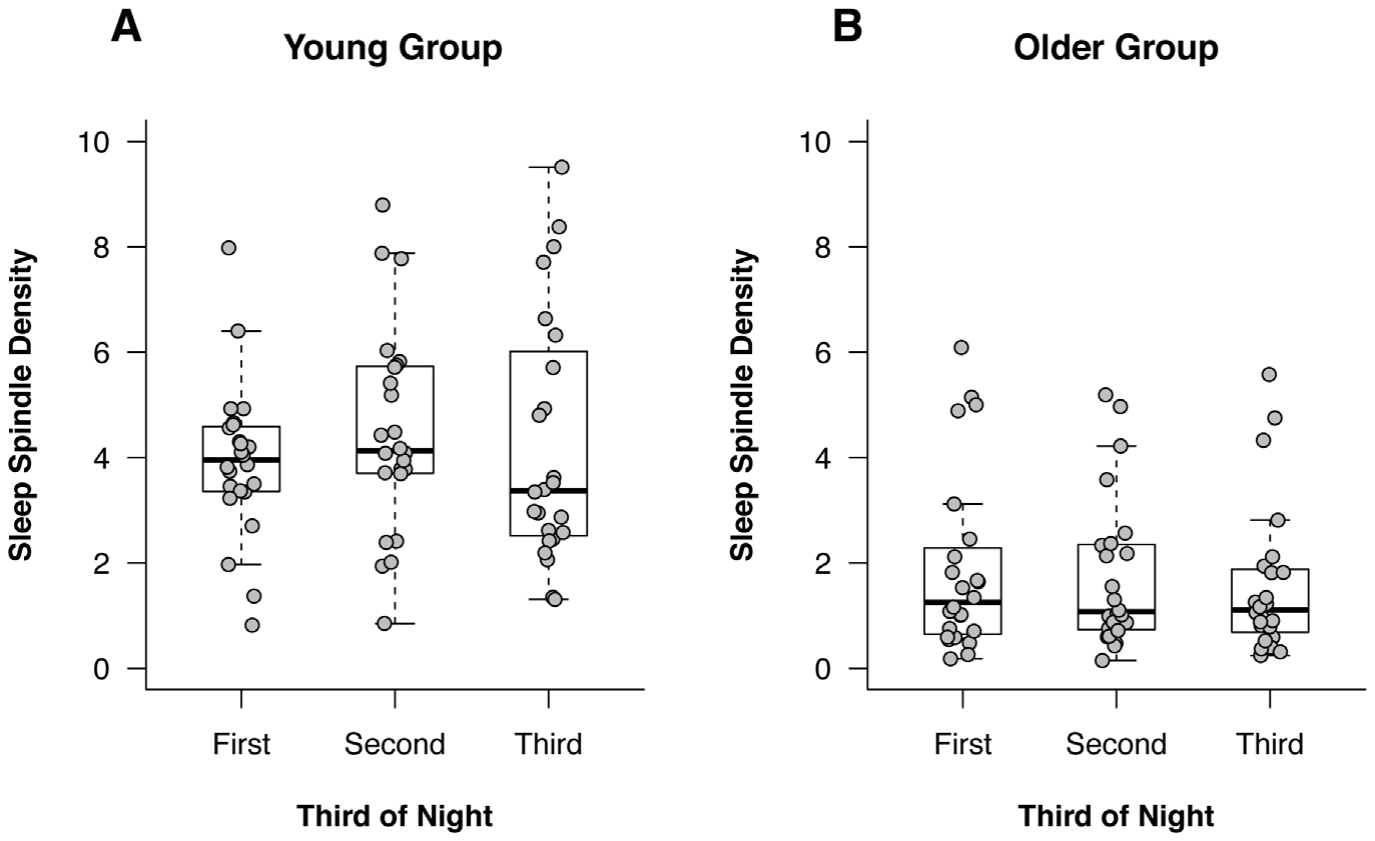
Sleep spindle density values across the night. The distribution of sleep spindle densities for each third of the night in Young adults (Panel A) and Older adults (Panel B) are shown. Both group-level (boxplots) and individual-level (dotplots) data are provided for each third of the night.

Change in spindle density across the night for both age groups are shown in Figure 2. The 2(Age Group)×3(Time) split-plot analysis for spindle density across the night revealed a significant main effect of Age Group, *F*(1,∞) = 31.69, *p*<.001; spindle density was significantly higher in the young adults (M_Rank_ = 98.00) than in the older adults (M_Rank_ = 46.00). The PS for this main effect was.85 (95% CI:.79 to.92). Neither the main effect of Time nor the Age Group×Time interaction were statistically significant, *p*>.05 for both. These results do not support the prediction that Stage 2 spindle density would increase significantly across the night in young but not older adults.

**Figure 3:**
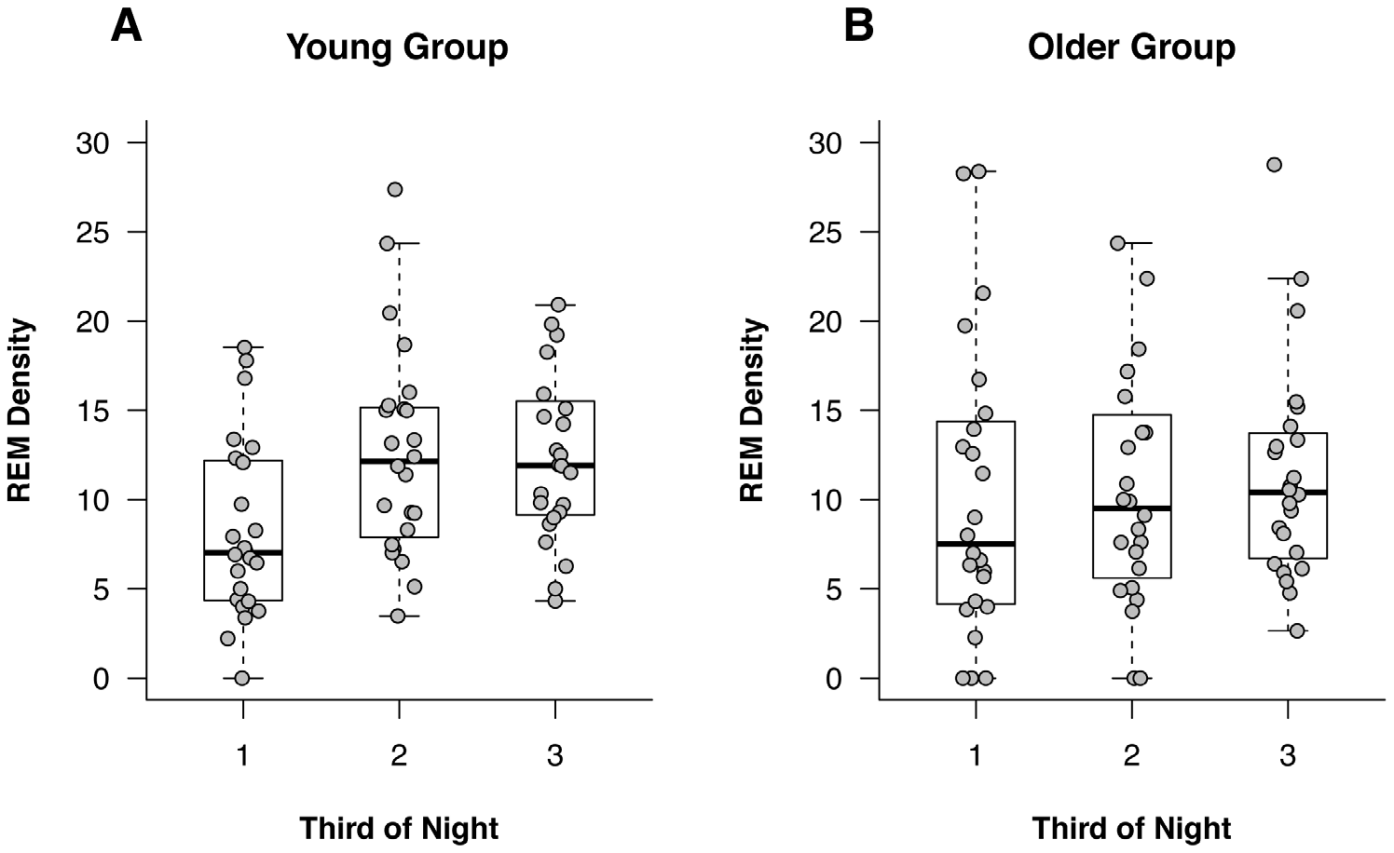
REM density values across the night. The distribution of REM densities for each third of the night in Young adults (Panel A) and Older adults (Panel B) are shown. Both group-level (boxplots) and individual-level (dotplots) data are provided for each third of the night.

Changes in REM density across the night for both age groups are shown in Figure 3. The 2(Age Group)×3(Time) split-plot analysis for REM density across the night revealed a significant main effect of time, *F*(1.95,∞) = 7.64, *p*<.001. Follow-up comparisons revealed that REM density in the Second Third of the Night (M_Rank_ = 55.00) was significantly higher than in the First Third of the night (M_Rank_ = 42.00), *F*(1,∞) = 10.90, *p*<.001, PS = .57 (95% CI:.53 to.61). REM density in the Last Third of the night (M_Rank_ = 56.17) was also significantly higher than in the First Third of the night (M_Rank_ = 40.82), *F*(1,∞) = 11.58, *p*<.001, PS = .56 (95% CI:.53 to.62). Although statistically significant, the PS values indicate that these effects are small. REM density did not differ between the Second Third (M_Rank_ = 47.50) and the Last Third (M_Rank_ = 49.50) of the night, *F*(1,∞) = 0.20, *p*>.05, PS = .49 (95% CI:.44 to.54). Neither the main effect of Age Group nor the Age Group×Time interaction were statistically significant, *p*>.05 for both, suggesting that changes in REM density over the course of the night are the same between young and older adults.

### Distribution Analysis

**Figure 4:**
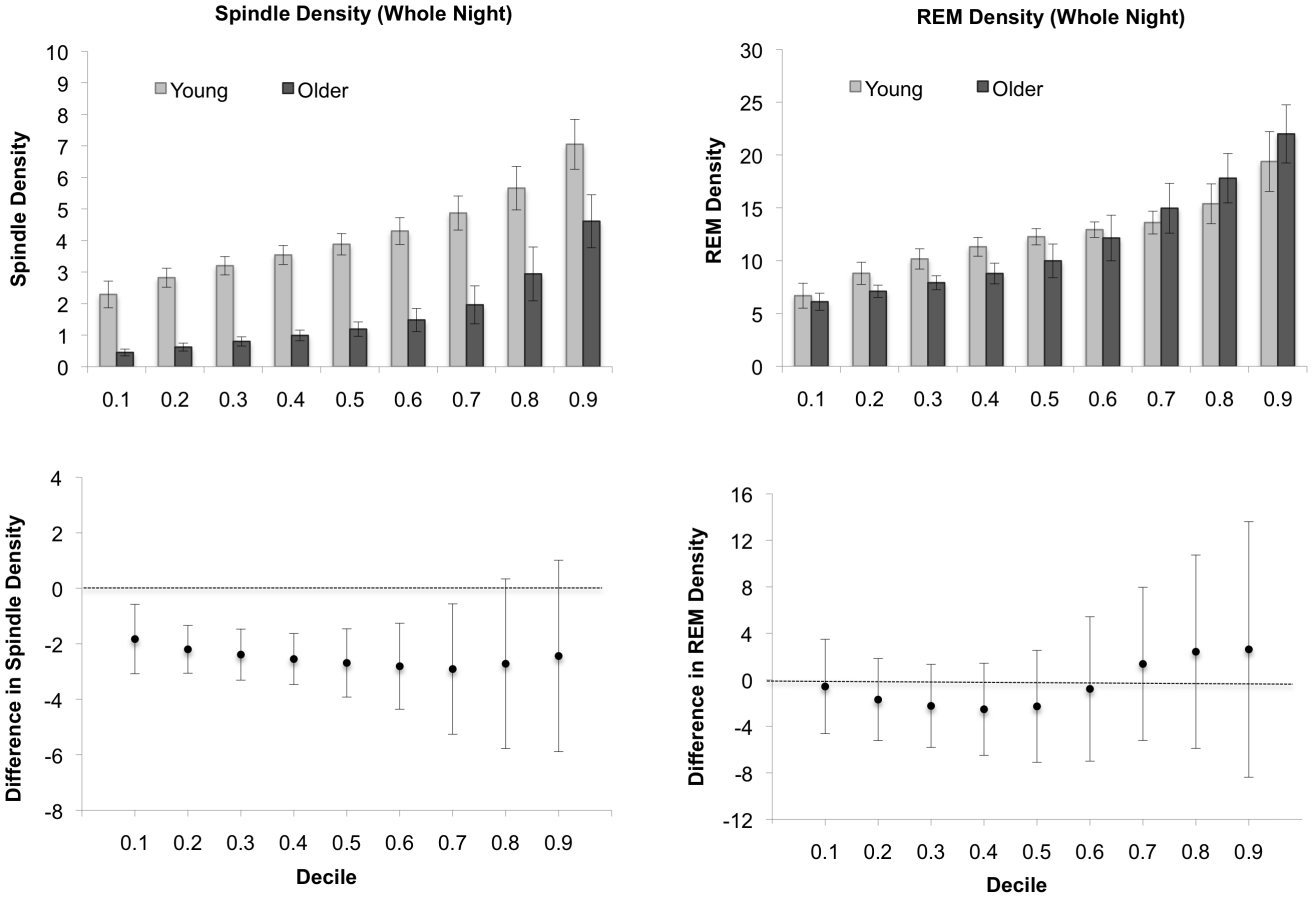
Distribution analysis for whole-night phasic events. Sleep spindle data are presented in the left panel and REM density data are presented on the right panel. The bar charts at the top are the Harrell-Davis estimates, and associated standard errors, for each decile in each age group. The charts at the bottom are difference scores, with corresponding 95% CIs for each decile (estimate for Older minus the estimate for Young); difference scores with CIs not containing zero (dashed line) are statistically significant at *p*<.05.

The distribution analysis for whole night sleep spindle and REM density is shown in Figure 4. The bar charts at the top of the figure are the Harrell-Davis estimates and standard errors for each decile in each age group. The plots at the bottom of the figure are the difference scores and associated 95% CIs for each decile. Sleep spindle density is significantly greater in the young adults at each decile except for the last two (0.80 and 0.90). As another indication of how large the age differences for spindle density are, the bar chart at the top of the figure clearly reveals that spindle density at the lowest decile (0.10) for the young is greater than all of the deciles for the older group up until the higher end of the distributions (0.80). It is also interesting to note the relative stability of the magnitude of effect across the distribution: the sleep spindle density estimates for the young adults are approximately 2 points greater at each decile. Thus, in this case, looking only at the group differences in the medians provides a fairly good indication of the magnitude of difference between the two groups throughout the distributions of values.

The 95% CIs crossed zero for each of the REM density decile comparisons, indicating that none of these differences are statistically significant. One common pattern seen for both sleep spindle and REM density is that the degree of variability is noticeably greater at the upper ends of the distributions. In the case of sleep spindles, it is this increased variability at the upper end that likely contributed the group differences not being statistically significant, as the magnitude of difference at these deciles is similar to the others.

**Figure 5:**
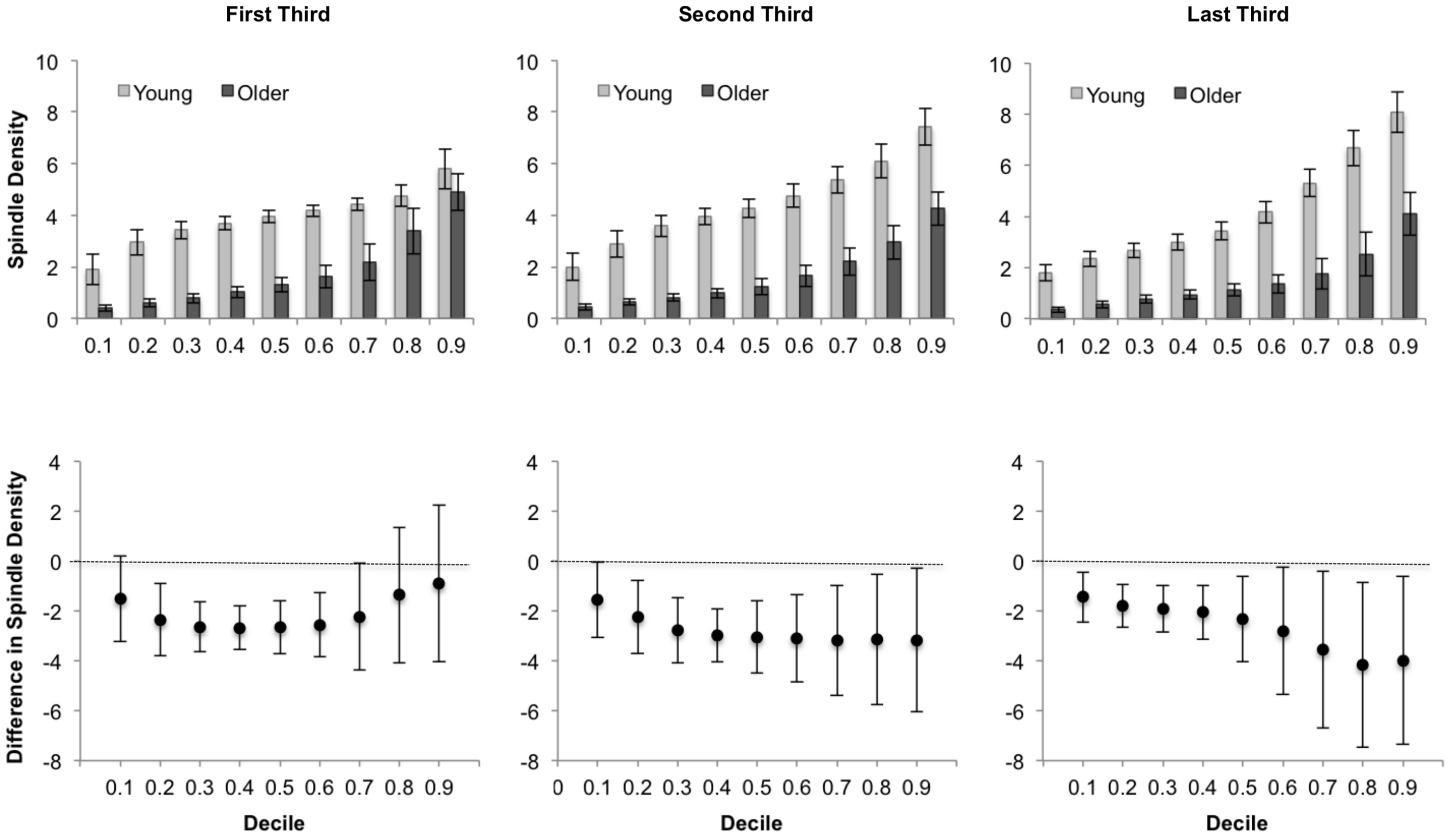
Distribution analysis for sleep spindle density across the night. Sleep spindle density data for the first (left panel), second (middle panel), and last (right panel) third of the night are shown. The bar charts at the top are the Harrell-Davis estimates, and associated standard errors, for each decile in each age group. The charts at the bottom are difference scores, with corresponding 95% CIs for each decile (estimate for Older minus the estimate for Young); difference scores with CIs not containing zero (dashed line) are statistically significant at *p*<.05.

The distribution analysis was performed for sleep spindle density during each third of the night (a similar analysis was not conducted for REM density as none of the age differences were statistically significant in the whole night analysis). The results are shown in Figure 5. The age differences during the first third of the night were statistically significant for the 0.20, 0.30, 0.40, 0.50 and 0.60 deciles. With the exception of the first decile (0.10) during the second third of the night, all of the remaining group differences were statistically significant. Perhaps the most noticeable trend that emerges from this analysis is that the magnitude of age differences is the largest and most statistically reliable for the middle of the distributions during the first third of the night, but as the night goes on, the magnitude of difference increases at the upper end of the distributions (deciles 0.70 through to 0.90). It is interesting to note that this pattern was not detected on the split-plot analysis based on ranked data, as the group by thirds interaction was not statistically significant. In addition, as with the whole night analysis, the largest amount of variability is observed at the upper end of the distributions.

### Correlation Analysis

The correlation between whole night sleep spindle and REM density was not statistically significant in the young adults, *rho* = 0.02 (95% CI: −0.42 to 0.45), or in the older adults, *rho* = 0.29 (95% CI: −0.19 to 0.68). In the young group, the correlation between sleep spindle and REM density during the first third, *rho* = 0.14 (95% CI: −0.50 to 0.31), second third, *rho* = 0.11 (95% CI: −0.38 to 0.50), and last third of the night, *rho* = 0.16 (95% CI: −0.31 to 0.58) were not statistically significant. Likewise, in the older adults, the correlations between sleep spindle and REM density during the first third, *rho* = 0.18 (95% CI: −0.25 to 0.55), second third, *rho* = 0.25 (95% CI: −0.16 to 0.59), and last third, *rho* = −0.10 (95% CI: −0.51 to 0.38) were not statistically significant.

## Discussion

The purpose of this study was to examine sleep architecture in young and older adults with a focus on sleep spindle and REM density. The results supported the prediction that sleep spindle density would be significantly higher in young adults compared to older adults. Contrary to our prediction, we did not find evidence of increases in spindle density across the night in the young participants. REM density did not differ between the two groups, but it did increase significantly across the night in both groups.

The finding of higher sleep spindle densities in the young group than in the older group is consistent with the literature [4]–[9]. The fact that there was no significant increase in spindle density across the night in the young adults is inconsistent with previous studies [6], [9], [10], and the reason for this finding is unclear. The lack of a significant increase in spindle density across the night in the older group, however, is consistent with these previous studies.

The finding of no age difference in REM density is consistent with some studies [5], [18], [19], but not with others [20], [21]. It is interesting to hypothesize that method of determining REM density may be an important factor here. Ehlers and Kupfer [45] reported no age differences in REM density with manual measures, but there were some age differences with automated measures. It seems that many of the studies that have used manual methods to measure REM density, including the present one, have found no significant age differences, but those reporting such differences have used automated methods. Clearly, more work needs to be done in this area. It was predicted that REM density would increase across the night in young adults, but this hypothesis was not supported. This result is inconsistent with two previous studies [19], [22], but it is consistent with the results reported by Feinberg [5]. He showed that REM density in both young and older adult age groups increased from the first to the second REM period but then leveled off from that point onward.

This was the first study, that we are aware of, that has examined two other important aspects of age differences in sleep architecture: variability and distributional differences. The Browne-Forsythe Levene type approach to comparing group variances revealed that the older adults showed significantly greater variability in SE and WASO than young adults. In terms of within-group comparisons, the findings revealed a similar pattern between the two age groups: percent of time spent in REM and Stage 2, TST, and SE were associated with the lowest degrees of relative variability in each age group, all having a CoD at or below 0.20. In both groups, the remaining CoD measures for percent of time spent in SWS, Stage 1, sleep spindle density, REM density, and WASO were remarkably larger than the first group, being more than double (i.e., greater than 0.40) for most measures. These findings suggest that the degree of variability is larger for some sleep measures (i.e., SWS, Stage 1, WASO, sleep spindle density, and REM density) than for others (i.e., REM, Stage 2, TST, and SE), and that this pattern is the same for the two age groups. These findings also suggest that variability for some sleep measures (e.g., SE and WASO) is significantly greater in older adults than in young adults. The results of this study may have important implications for theories of age-related changes in sleep architecture. For example, older adults spend significantly more time awake after sleep onset (WASO) and in Stage 2 sleep than young adults; however, variability in WASO, but not Stage 2 sleep is significantly greater in older adults than young adults. Future research on the mechanisms of age differences in the location (e.g., mean, median, etc) and scale (e.g., absolute and relative variability) of different sleep measures is clearly needed.

The present study revealed a novel and interesting aspect of age differences in sleep spindle density. It was shown that although the magnitude of age differences in whole night sleep spindle density was similar across the distributions, with density values being about two units higher in young adults for each decile, the pattern of such age differences changed across the night. In the first third of the night, the largest and most reliable age differences were observed in the middle of the distributions (deciles 0.30 to 0.60). However, as the night progressed, the largest age differences in sleep spindle density were observed in the upper end of the distributions (deciles 0.70 to 0.90). In fact, the magnitude of the age differences at the 0.80 and 0.90 deciles more than doubled between the first third of the night and the last third of the night. This pattern was not detected using the rank-based split plot analysis. One possible reason for this discrepancy is that this pattern of age differences is subtle in the sense that it is restricted to the upper end of the distributions, and the rank-based approach is not designed to detect such differences. It must also be kept in mind that the age differences for the majority of deciles were statistically significant, especially during the second and last third of the night. The distributional analysis reported here allowed a more detailed picture of age differences in spindle density to emerge, indicating a complex relationship between age, time-of-night and the inter-individual differences in spindle density. These results suggest that one single measure of sleep spindle density (e.g., the mean or median) is insufficient to adequately describe age differences in this measure across the night. These results may hold important implications for theories of age-related changes in sleep spindle density. For example, it has been suggested that age differences in sleep spindle density may explain the difficulties that many older adults have maintaining efficient and consolidated periods of sleep [4], [6], [7], and that they may also be related to age differences in intellectual or cognitive functioning [16], [17]. One of the reasons why the support for each of these hypotheses has been limited to date is that previous research has focused on single measures of location (e.g., the mean) and age differences across the distributions have not been taken into account. It may be the case, for example, that the relationship between sleep spindles and sleep maintenance/efficiency or cognition is different for individuals who have low or high levels of sleep spindles. Thus, physiological markers based on time-of-night changes in sleep spindles may help to predict outcomes such as impoverished sleep quality or cognitive decline with age. Future research on the implications of these results is needed, taking into account inter-individual variability.

Another major strength of the present study is that age differences in the density of sleep spindles and REMs were examined in the same participants. Typically, studies report on only one of these types of phasic events, making it difficult to interpret age differences because different samples are usually employed in different studies. In the present study it was shown that although there are large age differences in spindle density, there was no statistically significant difference in REM density. This pattern of results is consistent with those reported by Feinberg [5], the only other study that we are aware that also reported on these two phasic events in the same participants. We also found that spindle and REM density are not significantly correlated in either age group. This finding is of interest because both phasic events have been linked to cognitive function, and the lack of a significant correlation between them suggests a rather complex set of relations between spindles, REMs, and cognitive function in these two age groups, requiring further study.

In terms of limitations, we examined only one aspect of spindles and REMs – density. Part of the reason for this focus on density only was to choose a parameter on which these two phasic events could easily be compared. It would be useful to examine other characteristics of these phasic events in the future. For example, it would be interesting to examine spindle duration, amplitude, frequency, and scalp topography, as well as our analysis by slow and fast spindles [16]. For REMs, it would be interesting to examine the temporal nature of isolated REMs versus REM bursts as has been done in the literature [19].

The two age groups differed significantly on three of the four SDQ subscales. Older females and males scored significantly higher than the young groups on the Sleep Apnea and Periodic Limb Movement subscales. These age differences are not surprising as age-related increases in the prevalence of sleep disorder symptoms is a common finding in the literature [46], [47]. In fact, it would have been even more surprising if the two age groups did not differ on these subscales. The reason why the younger males scored significantly higher than the older males on the Psychiatric Sleep Disorder subscale is unknown. It is also important to point out that none of our participants scored above the published clinical cut-off scores for the SDQ subscales, and we also performed a limited overnight polysomnographic screening for possible sleep disorders. Thus, we are relatively confident that none of our participants had a sleep disorder. However, we cannot rule out the possibility that subclinical age differences on the SDQ subscales might have affected our results, as our nonparametric approach did not allow us to covary these variables.

In conclusion, the present study examined age differences in sleep architecture. In terms of sleep macroarchitecture, TST, SE, and amount of time in SWS were all significantly higher in young adults; in contrast, time spent awake after sleep onset (WASO) and in Stages 1 and 2 were significantly higher in older adults. The two age groups did not differ in the amount of time spent in REM sleep. Sleep spindle density was significantly higher in young adults than in older adults, but there was no significant age difference for REM density. In terms of time-of-night effects, there was a significant main effect of age group for sleep spindle density, with higher spindle densities in the young adults for each third of the night. There was also a significant main effect of time for REM density, such that REM density values increased significantly across the night in both groups. These results suggest that sleep spindle density is more affected by age than REM density. The present study also reported on two novel aspects related to age differences in sleep architecture. Although there were age differences in the degree of absolute variability (older adults had significantly larger variances than young adults for SE and WASO), a similar pattern was also observed in the two age groups: the four sleep measures with the lowest degrees of relative variability were the same and included time spent in REM and Stage 2 sleep, TST, and SE. The distributional analysis of age differences in sleep spindle density showed an interesting pattern across the night: Initially, the largest age differences were observed in the middle of the distributions, but as the night progressed, the largest age differences were seen on the upper end of the distributions. The results reported here have potential implications for the causes and functional implications of age-related changes in sleep architecture.
